# Farnesol inhibits translation to limit growth and filamentation in *C. albicans* and *S. cerevisiae*

**DOI:** 10.15698/mic2017.09.589

**Published:** 2017-09-04

**Authors:** Nkechi E. Egbe, Tawni O. Dornelles, Caroline M. Paget, Lydia M. Castelli, Mark P. Ashe

**Affiliations:** 1 Division of Molecular and Cellular Function, School of Biological Sciences, Faculty of Biology, Medicine and Health, The University of Manchester, Manchester Academic Health Science Centre, Michael Smith Building, Oxford Rd., Manchester, M13 9PT, United Kingdom.; 2 Current address: Department of Biological Sciences, Nigerian Defence Academy, PMB 2109, Kaduna, Nigeria.; 3 Current address: Sheffield Institute for Translational Neuroscience, University of Sheffield, Sheffield, S10 2HQ, United Kingdom.

**Keywords:** protein synthesis, translation control, farnesol, quorum sensing

## Abstract

*Candida albicans* is a polymorphic yeast where the capacity to switch between yeast and filamentous growth is critical for pathogenicity. Farnesol is a quorum-sensing sesquiterpene alcohol that, via regulation of specific signalling and transcription components, inhibits filamentous growth in *Candida albicans*. Here we show that farnesol also inhibits translation at the initiation step in both *Candida albicans* and *S. cerevisiae*. In contrast to fusel alcohols, that target the eukaryotic initiation factor 2B (eIF2B), farnesol affects the interaction of the mRNA with the small ribosomal subunit leading to reduced levels of the 48S preinitiation ribosomal complex in *S. cerevisiae*. Therefore, farnesol targets a different step in the translation pathway than fusel alcohols to elicit a completely opposite physiological outcome by negating filamentous growth.

## INTRODUCTION

The capacity to detect and respond to environmental change is essential for microorganism survival. This is especially true for opportunist pathogens like *Candida albicans*; where to initiate infection, the organism must adapt and persist in spite of host immune responses. Typically, *C. albicans* is a harmless commensal, yet in infected patients it causes various different conditions, from mucosal infections to life-threatening systemic infections 1.

Cell-cell signalling, particularly quorum sensing (QS), is a major focus of microbiological research. Farnesol is an acyclic sesquiterpene alcohol that represents the first QS molecule identified in eukaryotic microorganisms 2 where it causes a range of physiological effects 3. In* C. albicans*, farnesol inhibits the yeast to hyphal switch 2 to prevent colonization of different niche environments 4,5, it has antioxidant effects 6,7 and it inhibits transporters 8. In many species farnesol induces cellular death: for example in the fungal species, *Saccharomyces cerevisiae* 9, *Aspergillus nidulans* 10, *Penicillium expansum* 11, *Botrytis cinerea* 12 and even *C. albicans* under certain conditions 13. Equally, farnesol triggers cell death in mammalian cells 14 and can have antibacterial properties 15,16. In fact, farnesol was first discovered as a constituent of plant essential oils with antimicrobial activities 17.

Cellular responses to stimuli act via signal transduction pathways to regulate gene expression. In *C. albicans,* farnesol targets pathways like the Ras-PKA pathway that, via the transcription factors Efg1p and Czf1p and the repressor Tup1p, regulates gene expression 18. If a stimulus induces cellular stress, a transient inhibition of global protein synthesis is often observed, which further modulates the programme of gene expression to allow stress responsive gene expression programs to be initiated 19,20. Control of translation in this manner mostly occurs at the initiation stage in order to allow rapid and reversible management of gene expression.

Translation initiation is the assembly of an elongation competent 80S ribosome with an initiator methionyl-tRNA (Met-tRNA_i_^Met^) base paired via its anticodon loop to an mRNA Start codon 21. Highly conserved controls allow eukaryotic cells to globally reduce translation 19,20: a prominent example involves eIF2α kinases, like Gcn2p in *S. cerevisiae* 22,23. Gcn2p activation after amino acid starvation causes phosphorylation of the α subunit of eukaryotic translation initiation factor 2 (eIF2) 24. eIF2 is an essential GTP-binding protein that interacts with Met-tRNA_i_^Met^ to form a ternary complex (TC) that is competent for initiating translation 23. Phosphorylated eIF2 competitively inhibits the eIF2B-mediated guanine nucleotide exchange reaction on eIF2, reducing TC levels and translation initiation 23. However, specific mRNAs, such as yeast *GCN4,* continue to be translated under these conditions. *GCN4* encodes a transcription factor that regulates the expression of amino acid biosynthetic genes. This feedback regulatory circuit has proved a paradigm for studies on translation control 25. Similarly, *C. albicans* expresses a single eIF2α kinase, Gcn2p, which phosphorylates eIF2α in response to various stresses 26,27 and translational activation of *CaGCN4* also provides feedback regulation 28,29.

As well as indirect attenuation of eIF2B activity via phosphorylation of eIF2α, cells can also modulate eIF2B activity more directly. In mammalian cells, phosphorylation of eIF2B has been identified as an important regulatory mechanism 30. In addition, in both yeast and mammalian cells volatile anaesthetics appear to inhibit protein synthesis via eIF2B regulation 31,32. Moreover, in both *S. cerevisiae *and *C. albicans*, fusel alcohols, which are also characterised as quorum sensing molecules 33, have been shown to inhibit translation initiation in a mechanism that targets eIF2B but independently of the Gcn2p kinase or eIF2α phosphorylation 34,35,36.

Besides control via eIF2B, another regulated step in translation initiation is the mRNA selection phase 37. eIF4E and Pab1p select mRNA via interaction with the 5ʹ cap and 3ʹ poly(A) tail, respectively. eIF4G can interact with both eIF4E and Pab1p to form a closed loop complex that, via interactions with eIF3, eIF5 and eIF1, can recruit the small ribosomal subunit to form a 48S preinitiation complex 21. A variety of stress conditions have been shown to target these steps in the initiation pathway leading to transient reductions in translation to facilitate a switch to a new program of gene expression 19,20.

In this study, we show that as well as hampering various filamentation pathways, farnesol inhibits protein synthesis. This inhibition of translation occurs at the initiation step and most likely impacts upon the assembly of the 48S preinitiation complex. Intriguingly, this means two different quorum sensing agents, farnesol and fusel alcohols that have conflicting effects on filamentous growth, both inhibit translation initiation but by different mechanisms.

## RESULTS

### Farnesol inhibits growth and protein synthesis 

**Figure 1:**
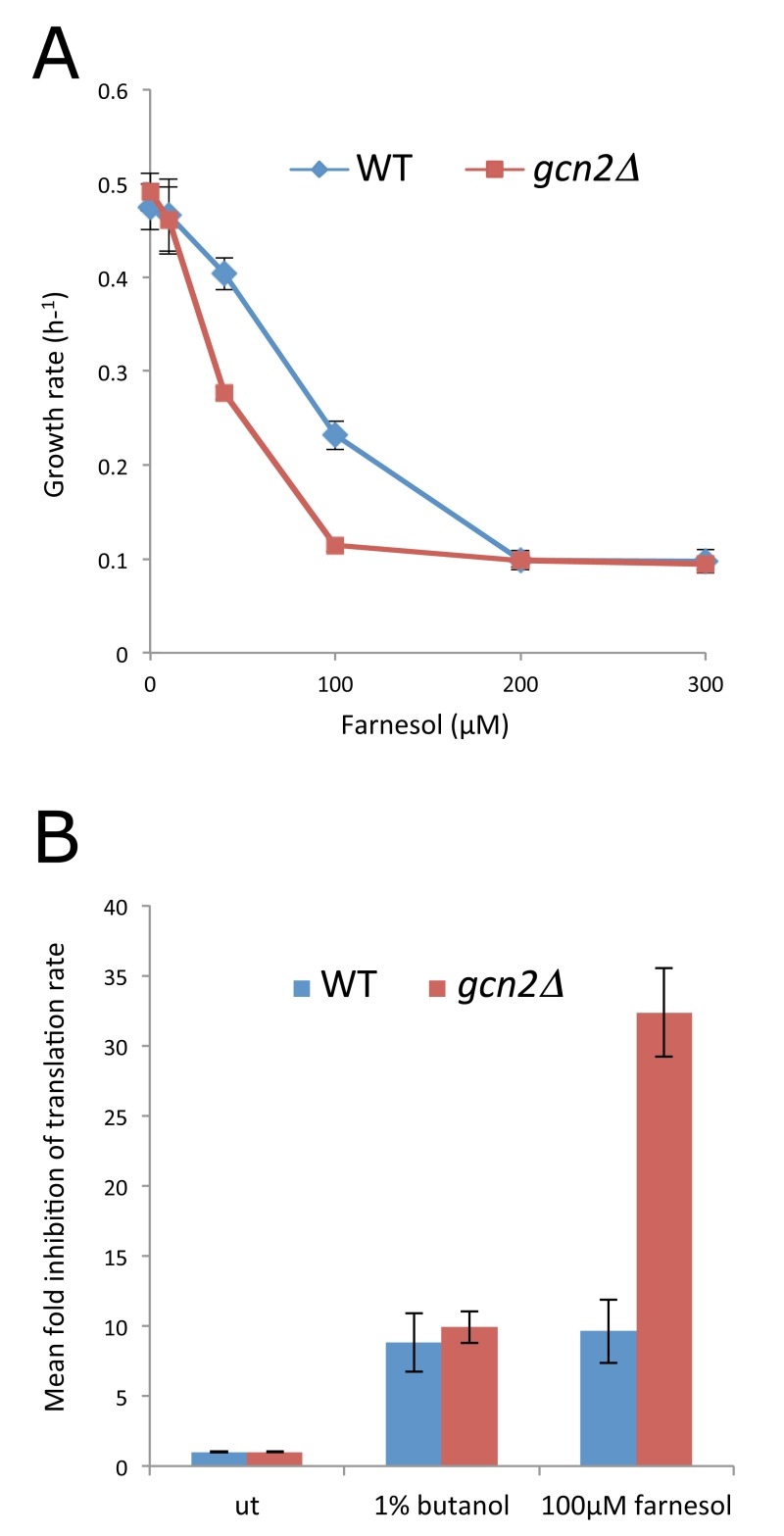
FIGURE 1: Farnesol inhibits growth and protein synthesis. **(A)** Growth rates for the CAI4 strain and the *gcn2*Δ mutant at various concentrations of farnesol as indicated. **(B)** Rates of radiolabelled methionine incorporation were measured for the CAI4 strain and the *gcn2*Δ mutant in untreated conditions (ut) or after 1% butanol or 100 µM farnesol treatment. These were used to calculate the fold inhibition for three biological replicates (error bars = SEM).

Farnesol, a eukaryotic QS molecule inhibits filamentous growth in both *S. cerevisiae* and *C. albicans *2, however, the concentration required varies according to the specific growth regime 38. Under the growth conditions used here, we found concentrations in excess of 100 µM farnesol inhibited the growth of *C. albicans *and an isogenic *gcn2∆* mutant (Fig. 1A). To study possible origins of the growth inhibition, the impact of farnesol on the rate of protein synthesis was monitored. The resulting [35^S^]-methionine incorporation data show that farnesol (300 µM) and butanol (2%) cause a 10-fold inhibition of protein synthesis in the CAI4 strain of *C. albicans* (Fig. 1B). Therefore, farnesol inhibits protein synthesis at very early stages after addition and this control could contribute to the growth inhibition observed.

**Figure 2:**
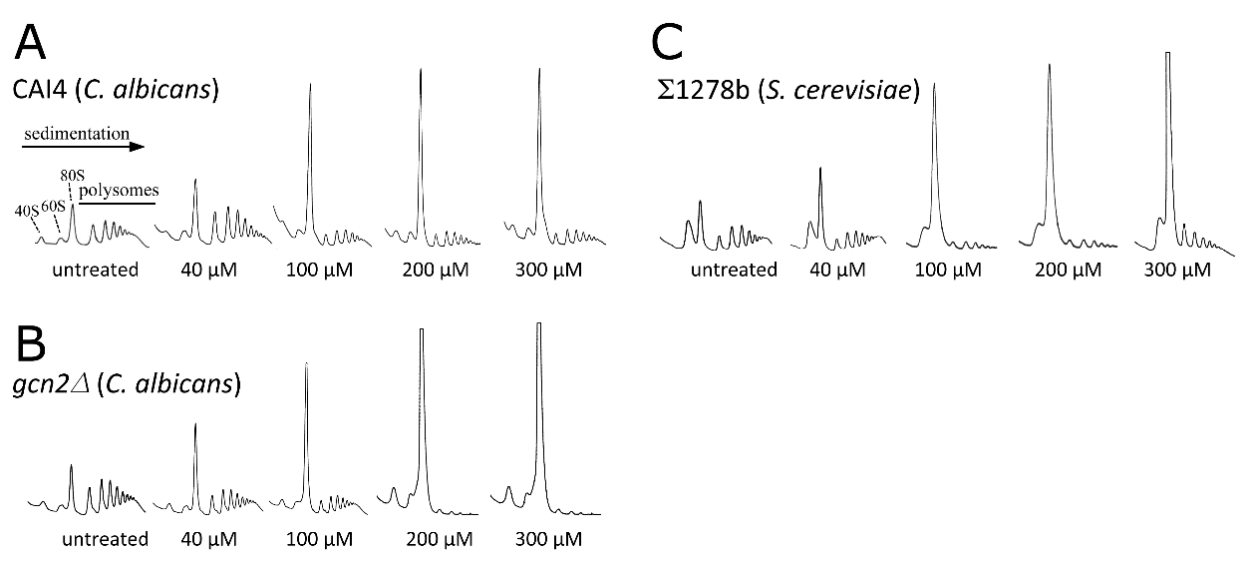
FIGURE 2: Translation initiation is inhibited by farnesol in a Gcn2p-independent manner in *C. albicans* and *S. cerevisiae*. Figure shows polysome analyses assessing the effect of farnesol on translation initiation in CAI4 **(A)** and *gcn2*∆ **(B)** strains of *C. albicans*, and the Σ1278b strain of *S. cerevisiae* **(C)**. Strains were grown in YPD and various concentrations of farnesol were added as indicated for 15 min prior to extract preparation. Extracts were sedimented on 15-50% sucrose gradients and the absorbance at 254 nm was continuously measured. The position of 40S, 60S and 80S peaks are labelled and the direction of sedimentation is noted.

To further investigate the stage of protein synthesis that is targeted by farnesol, polysome profiling was used, as this allows both the level of protein synthesis and the stage of regulation to be investigated 34,35,36. Analysis of polysome distribution for the *C. albicans* CAI4 strain revealed that increasing concentrations of farnesol caused a change in the polysome profile (Fig. 2A). The 80S peak increased dramatically and the polysome peaks were reduced. This change in profile is characteristic of an inhibition of translation initiation 34 and has been observed for many stresses 19. Similar results in terms of farnesol sensitivity were obtained for the Σ1278b strain of *S. cerevisiae*, where similar concentrations elicited the response across the two yeast species (Fig. 2C). It has been noted previously that the level of free 60S is particularly high for the Σ1278b, although this does not appear to impact upon its growth or its sensitivity to translational stress 39. The lowest farnesol concentration that caused a gross impact on polysome distribution for either *C. albicans* or *S. cerevisiae* was 100 µM (Fig. 2). This correlated well with the concentration that inhibited growth under the conditions used here (Fig. 1A) suggesting that the inhibition of translation initiation could be intrinsically connected to growth inhibition for farnesol.

The CAI4 strain of *C. albicans* used here is a commonly used lab strain that is auxotrophic in the uracil biosynthetic pathway by virtue of a homozygous deletion of the *URA3* gene 40. This mutation has previously been shown to alter a number of aspects of *C. albicans* physiology including adhesion and virulence 41. Therefore, a prototrophic strain of *C. albicans*, SC5314, was tested. Entirely analogous observations were made on the impact of farnesol on the growth (Fig. S1A) and translation (Fig. S1B) of this strain in response to similar concentrations of farnesol.

**Figure 3:**
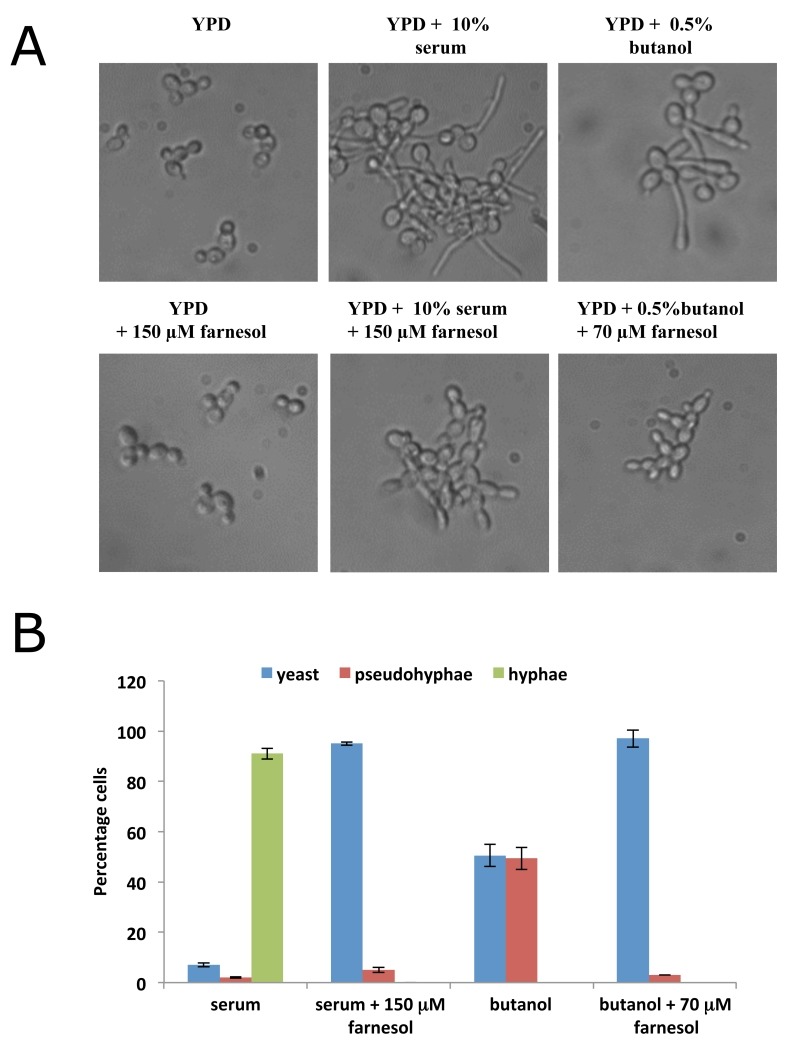
FIGURE 3: Farnesol represses morphological transition in *C. albicans*. **(A)** Overnight exponential cultures of the CAI4 strain were harvested, washed in water then incubated for 6 hours in different prewarmed media: YPD medium or YPD containing 150 µM farnesol; YPD containing 10% serum or YPD 10% serum with 150 µM farnesol; YPD containing 0.5% butanol or YPD 0.5% butanol with 70 µM farnesol. Cells were visualised via microscopy and photographed. **(B)** Cells from the above cultures were counted according to morphology using a cell counting chamber where pseudohyphae were scored if evidence of a restriction point between two cells was apparent, whereas hyphae were scored where elongated cells with no restriction points were viewed. Percentages are an average from three biological replicates (error bars = SEM).

Previous studies evaluating QS have established that *trans,trans-*farnesol is produced by *Candida* as a QS molecule to inhibit filamentation 2,42. Intriguingly, the *trans*,*trans* form and mixed stereoisomer preparations both impact upon translation initiation equally (Fig. S1). Indeed 40 µM of each is sufficient to induce a mild inhibition of protein synthesis and 100 µM leads to a robust inhibition (Fig. 1B). Therefore, in order to explore the mechanism by which farnesol inhibits translation initiation, over the course of the rest of our studies 100 µM farnesol was used as this concentration elicits robust inhibition of both growth and translation. However, it should be noted that lower concentrations of farnesol (e.g. 40 µM) can lead to subtle alterations in the polysome profile (Fig. 2, 3 and S1). This level of sensitivity to farnesol correlates well with earlier studies using similar growth conditions 38.

### *GCN2* is not involved in the inhibition translation initiation by farnesol

In terms of the mechanism of translational regulation, like *S. cerevisiae*, *C. albicans *harbours a single eIF2α kinase gene, *GCN2*, which is involved in the regulation of translation initiation in response to various stresses 26,27. Indeed in *S. cerevisiae*, *gcn2Δ* mutants are incapable of inhibiting translation initiation in response to specific stress conditions 34, which prevents cells from mounting an appropriate stress response. Therefore, the role of Gcn2p in the farnesol-dependent inhibition of translation initiation was assessed using a *gcn2*Δ mutant strain of *C. albicans *26. Previous observations using this strain show that translation initiation remains uninhibited early after amino acid starvation 29. In terms of the impact of farnesol on growth and translation, the *gcn2*Δ mutant strain is at least as sensitive as the wild type (Fig. 1A and B). In fact rather than the *gcn2*Δ mutant being resistant to farnesol in terms of translation, as might be expected if the Gcn2p kinase were involved in the control, the farnesol-treated *gcn2Δ* strain is even more inhibited than the wild type. Growth is inhibited at lower farnesol concentrations for the *gcn2*Δ mutant and methionine incorporation is inhibited up to 30-fold (Fig. 1A and B). Equally, a comparison of the polysome profiles shows that the *gcn2*Δ mutant exhibits somewhat greater sensitivity than the wild type mirroring the growth phenotypes (Fig. 2B). For instance, following treatment with 300 µM farnesol, greater polysome run-off is observed for the *gcn2*Δ mutant compared to the parent strain (*cf*. Fig. 2B with 2A). Overall, these results show that the Gcn2p kinase, that is a requirement for eIF2α phosphorylation and the subsequent regulation of translation initiation in response to a variety of stresses, is not required for the inhibition of translation initiation by farnesol in *C. albicans*; in fact the *gcn2*Δ mutant is more *sensitive* to treatment.

Fusel alcohols and other conditions that inhibit protein synthesis in *C. albicans* promote filamentous growth 36, whereas farnesol inhibits protein synthesis and *prevents* filamentation. An obvious query is whether the filamentation inducing signal generated by fusel alcohols can be overridden by farnesol or vice versa. Induction of filamentous growth is further complicated as different cues induce distinct forms of filamentation 43. For instance, fusel alcohols are characterised as inducing pseudohyphal growth 44 where elongated, ellipsoid yeast cells remain attached to one another via constricted septation sites leading to growth of a colony in a branched pattern 45. In contrast, serum addition elicits true hyphal growth 46, whereby cells are narrow, long, have parallel sides and no obvious constrictions points 45. In the presence of serum alone over 90% hyphal growth was observed and the addition of 150 µM farnesol blocked the yeast to hyphae switch (Fig. 3A and B). In contrast, the fusel alcohol, butanol, induces a much less robust effect whereby roughly 50% of cells exhibit pseudohyphal morphology. Here just 70 µM farnesol was sufficient to block any filamentous growth. These results show that farnesol competes with both serum and butanol, but the concentration of farnesol required to effect competition varies according to the strength of the filamentation signal (Fig. 3A and B). Curiously, even though both fusel alcohols and farnesol target protein synthesis, they are in competition with respect to their physiological impact on filamentous growth. Thus previous observations suggesting that the inhibition of protein synthesis favours filamentation 36 cannot be generalised across all conditions.

### Eukaryotic initiation factor 2B (eIF2B) is not regulated by farnesol

Fusel alcohols inhibit translation initiation in *S. cerevisiae* and *C. albicans* by targeting the guanine nucleotide exchange factor, eIF2B leading to reduced levels of the eIF2•GTP•Met-tRNA^i^ ternary complex 34,35,36.

**Figure 4:**
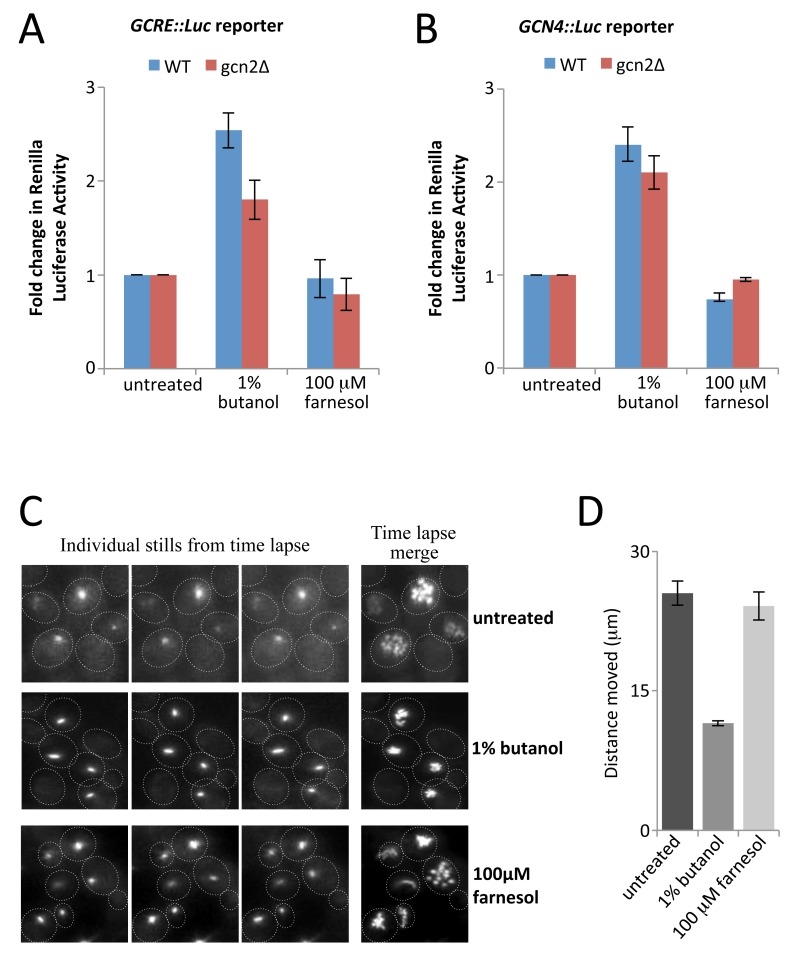
FIGURE 4: Farnesol does not induce *GCN4 *expression or effect the dynamics of the eIF2B body. **(A)** **and** **(B)** CAI4 and *gcn2*Δ strains bearing the *GCRE-Luc* (A) and *GCN4-Luc* (B) reporters were treated with 1% (v/v) butanol or 100 µM farnesol for 2 h, extracts were prepared, then renilla luciferase activity was measured relative to untreated. Error bars = ± SEM. **(C)** Images from time-lapse microscopy studies using an eIF2Bγ-GFP expressing *C. albicans* strain. The strains were incubated in media with 1% butanol, or 100 µM farnesol, or they were left untreated (UT) for 15 min as indicated. Each row contains three stills from a series of 25 images over a period of 2 min, as well as a merged image of all 25 stills, which serves to depict the total extent of 2B body movement. **(D)** Bar chart depicting the mean distance moved in μ over a 2-min period from 24 time-lapse experiments. Error bars, ±1 SEM.

Specific general control response reporters such as the *GCN4* reporter mRNA provide a sensitive indicator of changes in the ternary complex and are widely used to study translational regulation 25. A key observation that pointed towards eIF2B as a target for fusel alcohols was the demonstration that these reporters of ternary complex levels are translationally up-regulated 34,36. In order to assess this response after farnesol treatment, strains carrying two renilla luciferase reporters were used: the first contains five copies of the general control response element (GCRE), while the second harbours the* GCN4* promoter and leader region upstream 28,29. Using the parent and *gcn2∆* mutant strains bearing these reporters, the previous observation that 1% butanol elicits a non-Gcn2p dependent increase in the activity of *GCRE-Luc* and *GCN4-Luc* was confirmed (Fig. 4A and B). In stark contrast, farnesol elicits no significant increases of the *GCRE-Luc* or *GCN4-Luc* reporter expression (Fig. 4A and B) suggesting that farnesol does not alter ternary complex levels to activate the GCN response.

Previous studies on the localisation of eIF2B in organisms from *S. cerevisiae* to *Drosophila melanogaster* have defined a large cytoplasmic body called the eIF2B body (2B body) 35,36,47,48,49. Exposure of either *S. cerevisiae* or *C. albicans* cells to fusel alcohols reduces the dynamics of this 2B body in a manner that correlates with the sensitivity/ resistance of strains to alcohols 35,36. In order to ascertain whether farnesol also impacts upon 2B body dynamics, a *C. albicans* strain bearing GFP-tagged eIF2Bγ was used 36. Epiflourescence time-lapse microscopy experiments were performed by acquiring images of untreated, butanol treated or farnesol treated cells over a 2 min period. Movement of the eIF2B body across the images was tracked and the total distance (µm) moved was calculated. Quantitation of the average displacement shows that 1% butanol causes total eIF2B body movement to drop by approximately 50%. In contrast, in farnesol treated cells the 2B body moves to the same extent as in the untreated cells (Fig. 4C and D). This observation again suggests that the regulatory mechanism by which farnesol inhibits translation initiation is distinct from that of fusel alcohols and is not dependent upon eIF2B regulation or the alteration of ternary complex levels.

### Farnesol inhibits translation initiation by targeting 48S preinitiation complex formation

Translation initiation is controlled at other levels besides ternary complex formation and eIF2B. For instance, the interaction of mRNA with the 43S preinitiation complex, i.e. 48S preinitiation complex formation, can also be regulated 19,20,37. This process relies upon interactions between proteins that bind the mRNA and proteins associated with the 40S ribosomal subunit complex. One way to assess the factors present with the 40S ribosomal subunit is to perform immunoblotting on fractions collected from across sucrose density polysomal gradients. Formaldehyde cross-linking prior to cell lysis stabilizes protein factors in such complexes during the subsequent sedimentation and fractionation steps 50,51.

A limitation of such studies in *C. albicans* is that many antibodies against translation factors that are available for *S. cerevisiae *do not cross-react with *C. albicans* proteins (data not shown). Therefore, to further investigate the step in the translation pathway that is targeted by farnesol, investigations were undertaken in *S. cerevisiae.* The Ʃ1278b laboratory strain was selected, as like *C. albicans,* Ʃ1278b is diploid and can undergo morphogenetic switching to pseudohyphal growth 44. In order to validate the use of this strain, the effects of farnesol on growth and translation were cross-compared. Farnesol inhibits growth and translation initiation over a similar concentration range for the two yeasts and for other lab strains of *S. cerevisiae*, such as BY4741 (Fig. 2C; data not shown), so it seems likely that the translational responses to butanol and farnesol are mechanistically conserved across these species.

**Figure 5:**
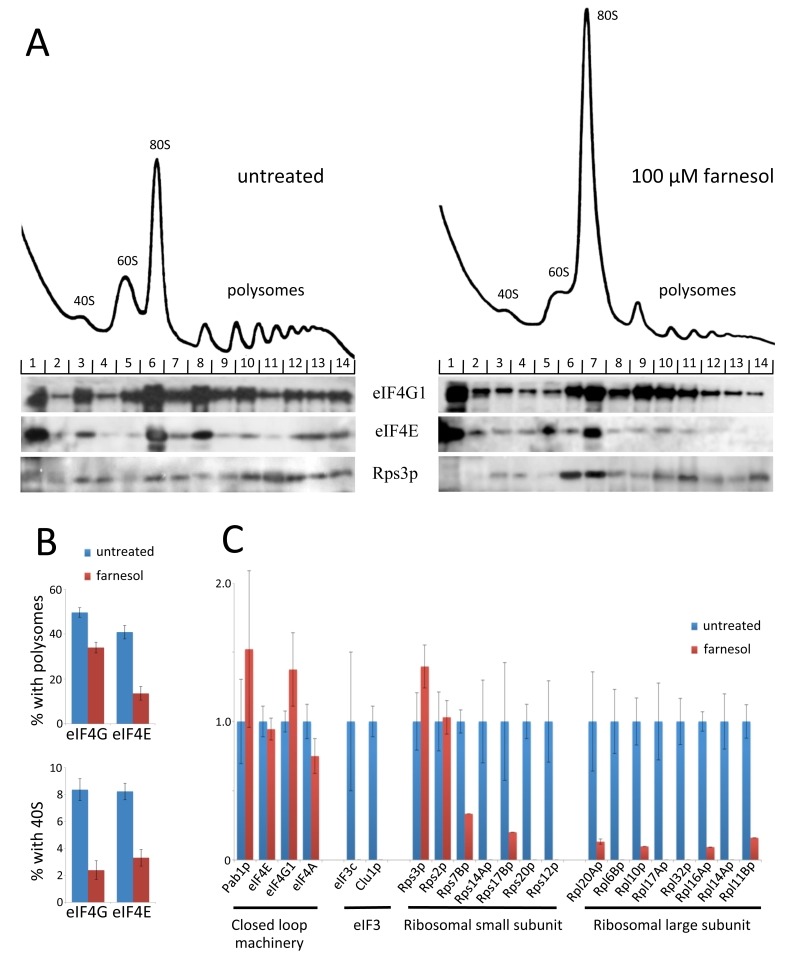
FIGURE 5: Farnesol affects the association of specific translation factors with the 48S pre-ribosomal complex. **(A)** Formaldeyhyde sucrose density gradient analysis on extracts from the MLY61 strain either treated with 100 μM farnesol for 15 min or untreated. Immunoblots on gradient fractions are shown below the traces and these were probed using antibodies against the indicated proteins. **(B)** Quantitation of the proportion of eIF4G and eIF4E present in the polysome fraction of the gradients before and after farnesol treatment. Quantitation of the proportion of eIF4G and eIF4E present in the 40S region of gradients before and after farnesol treatment. **(C)** Whole cell extracts and immunoprecipitation samples derived from the eIF4G1-TAP tagged strain treated with 100 μM farnesol or untreated were analysed via mass spectrometry. The plot shows the relative number of unique peptides that have been matched to the identified protein in the sample where the untreated was normalised to 1 to facilitate a direct comparison of the different translation factors.

Formaldehyde polysome analysis after farnesol treatment revealed an interesting effect in terms of the region of the gradient harbouring the 40S ribosomal subunit. This region not only contains free 40S ribosomal subunits but also the 48S ribosomal preinitiation complex where the 40S subunit is associated with mRNA and translation initiation factors i.e. an intermediate in the translation initiation process. A marker for this complex is the presence of translation initiation factors such as eIF4E and eIF4G that are specifically targeted to the mRNA rather than the 40S ribosomal subunit. The level of both eIF4G1 and eIF4E in the 40S region decreased dramatically after farnesol treatment (Fig. 5A, *cf*. fraction 3 untreated and fraction 3 treated). Quantitation confirmed that levels dropped from ~8-9% of total to 2-3% after farnesol treatment (Fig. 5B). Furthermore, both eIF4G and eIF4E are reduced in polysome regions and this likely reflects reduced levels of initiating ribosomes on mRNAs that are already being translated: although it should be noted that the scale of reduction is greater for eIF4E than eIF4G (Fig. 5B). This may relate to the fact that eIF4G can interact with RNA, Pab1p and other translation factors, whereas eIF4E is targeted to the mRNA cap. In sum, these data highlight the possibility that farnesol causes an alteration in protein-protein interactions that lie upstream of 48S complex formation.

In order to further investigate how farnesol treatment leads to eIF4G/eIF4E depletion from regions of the gradient, we undertook an immunopurification-mass spectrometry strategy using an eIF4G1-TAP tagged *S. cerevisiae* strain. In terms of the relative number of peptides observed in the immunopurified samples, peptides from known mRNA associated factors, such as eIF4G, eIF4E, eIF4A and Pab1p, were largely unaffected by farnesol (Fig. 5B). In contrast, peptides for other components of the translation machinery were reduced dramatically; including peptides for the ribosomal proteins, as well as subunits of eIF3, a translation initiation factor that is associated with the 40S ribosomal subunit. Overall, these data support a model where farnesol targets the formation of the 48S preinitiation complex to inhibit protein synthesis. The fact that farnesol targets a different step in translation to fusel alcohols, may mean that the impact of these agents and the stage targeted contributes to the opposite effects in terms of filamentous growth (Fig. 3). This is suggestive that translational control plays an important role in the physiological response of *C. albicans* to QS molecules.

## DISCUSSION

A range of alcohols or their derivatives can act as signalling molecules across yeast species 2,44,52,53. The data presented here combined with that in our previous studies 36 show that both butanol and farnesol inhibit protein synthesis at the translation initiation stage in *C*. *albicans. *Both are metabolites of *C. albicans* that act as signalling molecules, yet have opposing effects on morphological transition 2,36. Previously, we have shown in both* S. cerevisiae* and *C. albicans* that short chain alcohols regulate protein synthesis by targeting the guanine nucleotide exchange factor eIF2B 34,35,36.

eIF2B regulation plays a critical role in reprograming gene expression as part of the response to stress across different eukaryotic cells 23. For instance, eIF2 phosphorylation by eIF2α kinases, like Gcn2p in yeast, inhibits eIF2B in response to stresses such as amino acid starvation 24, purine starvation 54 and rapamycin treatment 55. However, while these stresses target eIF2B in a Gcn2p-dependent manner, the mechanism by which short chain alcohols target eIF2B in both *S. cerevisiae* and *C. albicans* is Gcn2p-independent 34,35,36. The *GCN4* reporter experiments and analysis of the eIF2B body in this study suggest that the longer chain sesquiterpene alcohol farnesol inhibits translation initiation in a mechanism that does not involve eIF2B regulation; either Gcn2p-dependent or independent.

Many studies have reported translational controls targeting steps upstream of 48S preinitiation complex formation. For instance, glucose starvation in *S. cerevisiae* causes a reorganisation of the closed loop mRNP translation complex, whereby eIF4A dissociates and the cosedimentation of eIF4E, eIF4G and Pab1p with ribosomal complexes is compromised 51. The small non-coding BC RNAs in neuronal cells target the eIF4A helicase to inhibit 48S preinitiation complex formation on structured mRNAs 56. Similarly, *Burkholderia* lethal factor 1, a toxin produced by *Burkholderia pseudomallei*, which causes the disease melioidosis, provokes a translational block via eIF4A 57. 48S complex formation can also serve as the targeted step when *specific* mRNAs are translationally regulated. For instance, miRNAs have recently been shown to inhibit target mRNA translation by impacting upon eIF4A2 activity 58. Therefore, a common translational regulatory mechanism that impacts upon the level of the 48S preinitiation complex is to target eIF4A activity.

In this study, we have investigated how farnesol effects different ribosomal complexes in *S. cerevisiae* using both formaldehyde-polysome analysis and immunoprecipitation followed by mass spectrometry. Both assays suggest that mRNA-associated translation factors (such as eIF4G, eIF4E and Pab1p) are associated less well with the ribosome and eIF3 following treatment with farnesol. Overall, the observed depletion of eIF4G and eIF4E from the 40S region of polysome gradients combined with the mass spectrometric analysis of eIF4G containing complexes lend support to a model where farnesol targets the formation of the 48S preinitiation complex to inhibit protein synthesis. This contrasts with the eIF2B dependent mechanism by which shorter chain alcohols target translation initiation.

Shorter chain alcohols and farnesol also differ in terms of their effects on morphological transitions in *C. albicans*. Short chain alcohols induce pseudohyphal growth in *C. albicans* whereas farnesol inhibits this process 2,44,53. Indeed we show that farnesol can impede the filamentation induced by a variety of triggers including short-chain alcohols. This is not without precedent, in *C. albicans* farnesol also blocks morphogenesis induced by the aromatic alcohol, tyrosol 59. One intriguing question is how both the shorter chain alcohols and farnesol can target a key ubiquitous process like protein synthesis, yet elicit distinct outcomes in terms of filamentous growth. This question drives at the fundamental physiological rationale for translational regulation in response to changing external conditions. Does the regulation constitute a knee-jerk reaction allowing the preservation of cellular resources by inhibiting the expression of the vast majority of mRNAs, or does the regulation serve a different purpose allowing specific mRNAs to be altered in their translation? In terms of farnesol, we show that translation is mildly inhibited at 40 µM farnesol and robustly down-regulated at 100 µM farnesol. Various *Candida* strains produce farnesol up to a concentration of ~60 µM 60, which would appear to favour the option where translation of a specific subset of mRNAs is altered. Evidence from a number of systems including the induction of *GCN4* translation via amino acid starvation 25 would also favour this option. Under such a scenario, if two stresses impact upon different stages of translation initiation, they might alter the translation of different subsets of mRNA. We have previously observed evidence for such effects in *S. cerevisiae*, where fusel alcohols and amino acid starvation alter translational reprograming to allow continued translation of different cohorts of mRNAs 61. With this in mind, we envisage that for farnesol and fusel alcohols, mRNAs encoding pro and anti-filamentation factors might be prominent in a set that are differentially regulated at the translational level. Such effects would also be integrated with well-defined transcriptional controls, especially for farnesol 18, to produce very different phenotypic outcomes.

## MATERIALS AND METHODS

### Media and growth conditions 

The strains in Table 1 were grown and maintained as described previously 36. Butanol and farnesol were routinely added for 15 min at the concentrations stated. Unless otherwise stated *trans, trans*-farnesol was used. Tolerance was assessed by adding butanol (0.5%, 1% and 2%) or farnesol (40 µM, 100 µM, 200 µM and 300 µM) to strains at OD_600_ 0.1 and then testing growth.

**Table 1:** Strains used in this study.

***C. albicans* strains**		
**Strain**	**Genotype**	**Source**
CAI4	*ura3::λimm434/ura3::λimm434*	A. Brown
CAI8	*ura3::λimm434/ura3::λimm434 ade2::hisG/ade2::hisG*	A. Brown
*gcn2*Δ	*ura3::λimm434/ura3::λimm434 gcn2::hisG/gcn2::hisG*	A. Brown
SC5314	Prototroph	C. Grant
yMK2313	*ura3::λimm434/ura3::λimm434 GCD1-GFP::NAT/GCD1*	Ashe lab
CY2383	*ura3::λimm434/ura3::λimm434 ADE2::GCRE-rLUC/ade2::hisG*	C. Grant
CY2511	*ura3::λimm434/ura3::λimm434 ADE2::GCN4-rLUC/ade2::hisG*	C. Grant
CY2387	*ura3::λimm434/ura3::λimm434 gcn2::hisG/gcn2::hisG ADE2::GCRE-rLUC/ade2::hisG*	C. Grant
CY2492	*ura3::λimm434/ura3::λimm434 gcn2::hisG/gcn2::hisG ADE2::GCN4-rLUC/ade2::hisG*	C. Grant
		
***S. cerevisiae* strains**		
**Strain**	**Genotype**	**Source**
MLY61(Σ1278b)	*MATa/MATα ura3-52/ura3-52*	J. Heitman
yMK2197	*MATa his3*Δ*1 leu2*Δ*0 met15*Δ*0 ura3*Δ*0 HIS3*	Open biosystems
yMK2084	*MATa his3*Δ*1 leu2*Δ*0 met15*Δ*0 ura3*Δ*0 TIF4631-TAP::HIS3*	Open Biosystems

### Morphogenesis assays

Exponential cultures were harvested, washed in water then re-inoculated into media with 0.5% butanol, 10% serum, 0.5% butanol-70 µM farnesol or 10% serum-150 µM farnesol. Filamentation was assessed microscopically as previously described 36.

### Analysis of polysomes and other translation assays

Exponential strains were incubated with butanol/ farnesol for 15 min then treated with cycloheximide: 1 mg/ml (*C. albicans*) or 0.1 mg/ml (*S. cerevisiae*). Extracts were prepared then polysome analysis and fractionation were carried out as previous 36. Formaldehyde polysome analysis was performed as described previously 50,51. Immunoblots were probed with antibodies to yeast eIF4G, eIF4E and Rps3.

[35^S^]-methionine incorporation assays were conducted by adding 60 ng/ml methionine, where 0.5 ng/ml was [35^S^]-methionine (PerkinElmer), to exponential untreated or farnesol/ butanol treated cultures in synthetic complete dextrose (SCD) medium lacking methionine. Samples (1 ml) were taken at the indicated times and processed as described previously 36.

For the Luciferase reporter assays 29, lysates were prepared from exponential untreated or farnesol/ butanol treated cultures in RLUC buffer (0.5 M NaCl, 0.1 M K_2_HPO_4_, 1 mM Na_2_EDTA, 0.6 mM sodium azide, 1 mM phenylmethylsulfonyl fluoride, 0.02% bovine serum albumin). 1.25 μM coelentrazine h (Promega) was added to the extracts to initiate the reaction, and activity was measured using a GloMax 20/20 luminometer (Promega). Luciferase activity (RLU) is expressed as relative luminescence per 10 s/mg protein.

For studies on the 2B-body 35,36, real-time 2D deconvolved projections were generated via continuous z-sweep acquisition on a Delta Vision RT microscope (Applied Precision, Isaaquah, WA) with an Olympus 100× 1.40 NA DIC oil PlanApo objective (Melville, NY) and Roper CoolSnap HQ camera (Tucson, AZ) with Applied Precision Softworx 1.1 software for fast visualisation of all planes with minimal fluorescent bleaching. Images were acquired every 5 s over a 2 min period, and ImageJ (http://rsb.info.nih.gov/ij; NIH) was used to track 2B body movement and calculate the mean total distance using at least 24 individual tracking experiments per condition.

### Affinity Purification and mass spectrometry 

For the eIF4G1-TAP purification, protein extracts were bound to IgG columns eluted with a TAP peptide, then samples were isolated from SDS PAGE gel slices 62. Dried gel pieces containing the whole protein sample were digested using 100 ng trypsin and analysed by LC-MS/MS using an UltiMate® 3000 Rapid Separation liquid chromatography (Dionex Corporation, Sunnyvale, CA) coupled to a LTQ Velos Pro mass spectrometer (Thermo Fisher Scientific, Waltham, MA). Data were searched using Mascot (Matrix Science UK), against the Uniprot database with *S. cerevisiae* selected. Data were validated and further processed using Scaffold (Proteome Software, Portland, OR).

## SUPPLEMENTAL MATERIAL

Click here for supplemental data file.

All supplemental data for this article are also available online at http://microbialcell.com/researcharticles/farnesol-inhibits-translation-to-limit-growth-and-filamentation-in-c-albicans-and-s-cerevisiae.
