# Supplementary material for: Farnesol inhibits translation to limit growth and filamentation in C. albicans and S. cerevisiae
Source: Microb Cell. 2017 Sep 4;4(9):294–304. doi: 10.15698/mic2017.09.589 (PMC5597792; doi:10.15698/mic2017.09.589)
Supplement: Supplementary file 1 [file mic-04-294-s01.pdf]

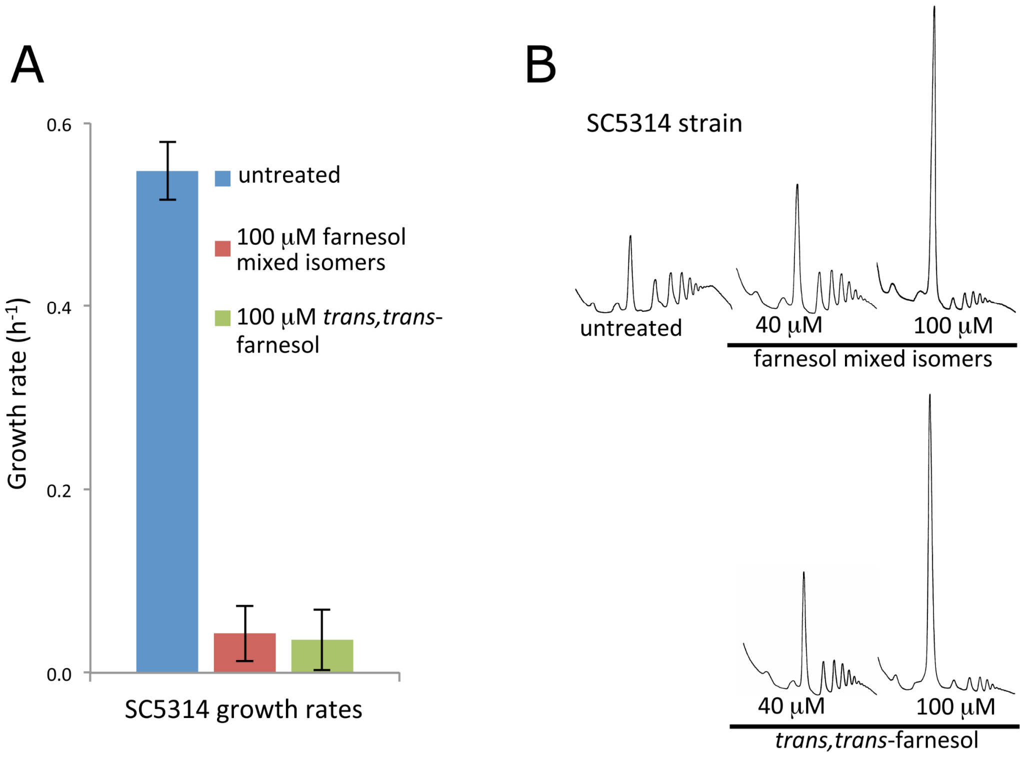

FIGURE S1: Both farnesol (mixed isomers) and *trans,trans*-farnesol inhibit prototrophic *C. albicans* strains at the level of growth and translation. (A) Growth rates for the SC5314 strain of *C. albicans* show similar inhibition of growth by farnesol to the CAI4 (error bars = SEM). **(B)** Polysome profiles show the impact of mixed isomer farnesol and *trans,trans*-farnesol on translation initiation in the SC5314 strain. SC5314 exhibits a similar inhibition profile to CAI4 (see Fig. 1) and both forms of farnesol exhibit similar efficacy.
